# Antimicrobial Photodynamic Therapy in Combination with Nystatin in the Treatment of Experimental Oral Candidiasis Induced by *Candida albicans* Resistant to Fluconazole

**DOI:** 10.3390/ph12030140

**Published:** 2019-09-18

**Authors:** Karem Janeth Rimachi Hidalgo, Juliana Cabrini Carmello, Cláudia Carolina Jordão, Paula Aboud Barbugli, Carlos Alberto de Sousa Costa, Ewerton Garcia de Oliveira Mima, Ana Claudia Pavarina

**Affiliations:** 1 Department of Dental Materials and Prosthodontics, School of Dentistry, São Paulo State University (Unesp), Araraquara, Rua Humaitá, 1680, Araraquara 14801-903, SP, Brazil; krimachi_24@hotmail.com (K.J.R.H.); cabrini.juliana@gmail.com (J.C.C.); claudia.jordao@unesp.br (C.C.J.); pabfarma@yahoo.com.br (P.A.B.); ewerton.mima@unesp.br (E.G.d.O.M.); 2 Department of Physiology and Pathology, School of Dentistry, São Paulo State University (Unesp), Araraquara, Rua Humaitá, 1680, Araraquara 14801-903, SP, Brazil; casouzac@foar.unesp.br

**Keywords:** photodynamic therapy, *Candida albicans*, antifungals

## Abstract

Background: It has been demonstrated that azole-resistant strains of *Candida albicans* have a greater resistance to antimicrobial photodynamic therapy (aPDT) when compared to their more susceptible counterparts. For this reason, the present study evaluated the efficacy of aPDT, together with nystatin (NYS), in the treatment of oral candidiasis in vivo. Methods: Mice were infected with fluconazole-resistant *C. albicans* (ATCC 96901). To perform the combined therapy, aPDT, mediated by Photodithazine (PDZ) and LED light, was used together with NYS. The efficacy of the treatments was evaluated by microbiological, macroscopic, histopathological and Confocal Scanning Laser Microscopy analyses of the lesions. The expression of p21 and p53, proteins associated with cell death, from the tongues of mice, was also performed. Results: The combined therapy reduced the fungal viability by around 2.6 log_10_ and decreased the oral lesions and the inflammatory reaction. Additionally, it stimulated the production of p53 and p21. Conclusions: The combined therapy is a promising alternative treatment for oral candidiasis induced by *C. albicans* resistant to fluconazole.

## 1. Introduction

*Candida* spp. are commensal constituents of the oral microbiota and gastrointestinal tract in healthy individuals [1]. Under certain circumstances, such as a pH imbalance, nutritional changes, the prolonged use of antibiotics, and changes in the immune system caused by infection or immunosuppressive therapy [2,3], these microorganisms may act as opportunistic pathogens, proliferating and promoting the development of superficial fungal diseases, for instance, oropharyngeal candidiasis (OPC) [4]. OPC may progress to bloodstream infections, which have been an important cause of morbidity and mortality, especially in hospitalized and immunocompromised patients [5]. Moreover, the development of intrinsic and acquired antifungal resistance in the *Candida* species has directed the scientific community to search for new treatments, aiming to improve the outcome of patients with these infections, for instance, antimicrobial photodynamic therapy (aPDT).

The aPDT action includes a photosensitizing agent (PS), light irradiation with a specific wavelength, and oxygen [6]. When these components come in contact with one another, reactive oxygen species (ROS), which are responsible for the promotion of cell inactivation, are generated [7,8]. It has been shown that the efficacy of aPDT is related to the type of PS used [9,10]. Photodithazine^®^ (PDZ) is a derivative of chlorine e6, a second-generation PS, which is soluble in water and produces a high quantum yield of singlet oxygen [8]. This PS presents a high band of absorption in the red area of the electromagnetic spectrum (650 nm to 680 nm), which allows it to penetrate deeper into biological tissues, improving the action of aPDT [8,11]. In vitro investigations have pointed out that aPDT mediated by PDZ is a promising technique for inactivating clinical isolates and single and multispecies biofilms of *Candida* [12,13,14]. An in vivo study observed that one application of aPDT mediated by PDZ decreased the *C. albicans* viability by 4.36 log_10_ [15]. In another study, a single session of aPDT mediated by PDZ reduced the cell viability of fluconazole-resistant *C. albicans* in mice with experimental oral candidosis by 1.96 log_10_ (OC) [16]. Recently, a modified protocol for experimental OC in mice was shown to enable the aPDT treatment and monitoring of oral lesions [17]. Five consecutive applications of aPDT mediated by PDZ or antifungal Nystatin resulted in reductions of 3 and 3.2 logs_10_, respectively, in the cell viability. In addition, macroscopic resolution of lesions was detected in all animals treated with aPDT, while the animals that received Nystatin showed a partial remission of oral lesions [17]. Based on the above-mentioned studies, aPDT mediated by PDZ demonstrated satisfactory results in the inactivation of *Candida* spp. and in the treatment of experimental OC [12,13,14,15,16,17]. However, the eradication of this pathogen has not been shown, and the effectiveness of aPDT in the treatment of OC induced by *C. albicans* resistant to fluconazole has not been evaluated.

Regarding the resistance of *Candida* spp., new strategies have been suggested for the inactivation of this pathogen, with the aim of improving the effectiveness of the treatments, for instance, the combination of aPDT with the traditional therapies [18]. The combination of aPDT mediated by Curcumin (CUR) with the antifungal fluconazole was effective in inactivating the biofilm of *C. albicans* growth and their planktonic cultures after 18 h [19]. The authors observed an improvement of the efficacy of the treatment, with biofilm metabolism being reduced from 100% to 5%. In another study, aPDT mediated by 5-aminolevulinic-acid associated with Gentamicin reduced the metabolic activity of biofilms of *Staphylococcus epidermidis*, *Staphylococcus aureus* and *Staphylococcus haemolyticus* by 30% [20]. In the above-mentioned investigations, only one application of the combined therapy was performed. However, it is important to mention that only in vitro studies using the combined therapy were evaluated.

It has been reported that aPDT treatment is related to necrosis, apoptosis and autophagy-associated cell death [21,22,23]. Some proteins, such as p53 and p21, were involved in these processes. The p53 protein exhibits several functions in cell biology in response to various stress factors [24]. There are well-described pathways for the p53 functions, for example, in the regulation of cell cycle control, the induction of apoptosis, autophagy, and DNA damage response [24,25,26]. In addition, p53 directly regulates the production of p21, another important protein related to cell cycle control, senescence and response to DNA damage [27]. Cellular photosensitization can also occur by p53-dependent and p53-independent pathways [24]. Little is known about the roles of p53 e p21 in cell death after treatment with aPDT.

Therefore, the purpose of the present investigation was to evaluate the efficacy of aPDT mediated by PDZ, together with the antifungal nystatin, in the treatment of induced OC in mice infected with *C. albicans* resistant to fluconazole. Reduction of the fungal load, macroscopic lesions, and histopathological and confocal analysis of the tongues of the mice were performed immediately and after 7 days of the treatments. Additionally, the expression of proteins p21 and p53 from the animals’ tissue was evaluated.

## 2. Results

### 2.1. Microbiological Evaluation

**Figure 1:**
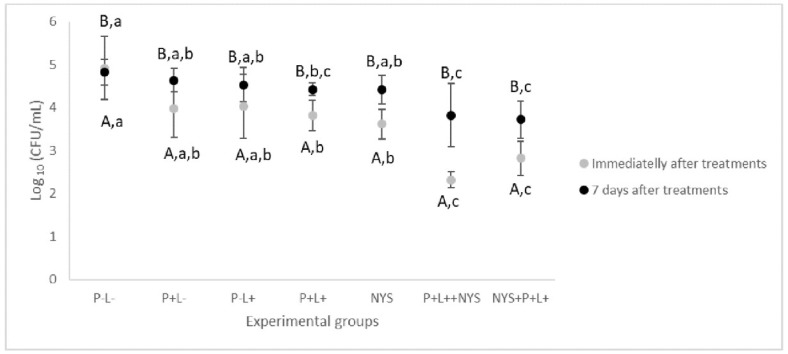
Mean values and standard deviation of log_10_ (CFU/mL) of fluconazole-resistant *C. albicans* recovered from animals from all the experimental groups immediately and after 7 days of the treatments. The uppercase letters (A and B) denote statistical differences between the time intervals evaluated (*p* ≤ 0.05) and the lowercase letters (a, b, and c) denote statistical differences between the experimental groups (*p* ≤ 0.05, two-way ANOVA).

Figure 1 shows that immediately after the treatments, the animals treated with aPDT followed by nystatin (P+L+NYS+ group) showed the highest values of log_10_ reduction (CFU/mL of fluconazol-resistant *C. albicans*) in comparison with the negative treatment control (P-L- group) (*p* ≤ 0.0001), which was equivalent to 2.6 log_10_. The inversion of the order of application of the combined therapy (animals treated with nystatin first, followed by aPDT—the NYS+P+L+ group) also reduced the cell viability by 2.1 log_10_ (*p* ≤ 0.0001). The animals who received only aPDT mediated by PDZ (P+L+ group) and nystatin (NYS group) presented a similar reduction in cell viability, equivalent to 1.3 and 1.1 log_10_ (*p* = 0.912), respectively, and were statistically different from the P-L- group (*p* = 0.004, and *p* = 0.013, respectively) and the combined therapy (P+L+NYS+ and NYS+P+L+) (*p* ≤ 0.0001). The isolated application of light (P-L+ group) or PDZ (P+L- group) yielded statistically similar results to those of the P-L- group (*p* ≥ 0.05).

Regarding the period of 7 days after the treatments, the data related to the combined therapy were statistically similar to each other and different from the P-L- group, with a reduction of the fungal viability of 1.0 and 1.1 log_10_ (*p* = 0.024, and *p* ≤ 0.001, for the P+L+NYS+ and NYS+P+L+ groups, respectively) (Figure 1). The P+L- and P-L+ groups were similar to the P-L- group (*p* ≥ 0.05). The P+L+ group presented values that were statistically similar to those of the P+L+NYS+ (*p* = 1.000) and the NYS+P+L+ (*p* = 0.255) groups.

### 2.2. Macroscopic Evaluation of Lesions

**Figure 2:**
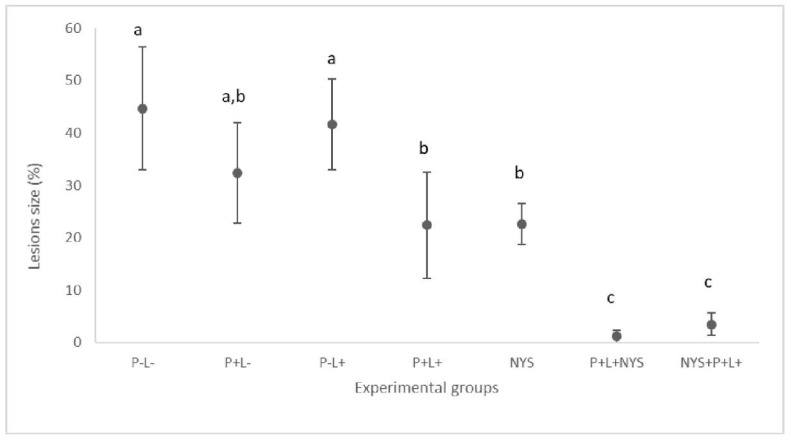
Mean values and standard deviation in percentages (%) of the lesion size (with the size of the patches) on the dorsum of the tongues of all the evaluated animals after 24 h of treatment. The letters a, b and c denote statistical differences between the groups (*p* ≤ 0.05).

The combined therapy significantly reduced the oral lesions by 97.34% and 92.23% for the P+L+NYS+ and NYS+P+L+ groups, respectively, when compared to the P-L- group (*p* ≤ 0.0001). Regarding the P+L+, NYS and P+L- groups, the reductions in the oral lesions was statistically different from the P-L- group (*p* ≤ 0.05) (Figure 2). These results were obtained after 24 h of treatment.

**Figure 3:**
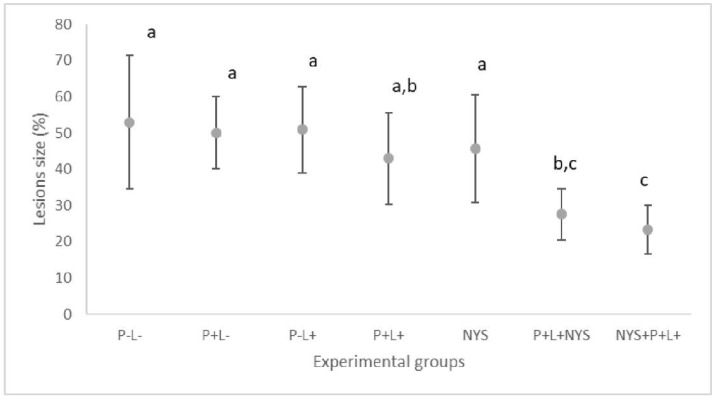
Mean values and standard deviation in percentages (%) of the lesion size (with the size of the patches) on the dorsum of the tongues of all the evaluated animals after 7 days of treatment. The letters a, b and c denote statistical differences between the groups (*p* ≤ 0.05).

After seven days of therapies, the macroscopic evaluation revealed that animals treated with the combined therapy presented a significant reduction in the oral lesions, which corresponded to 47.88% and 55.23% for the P+L+NYS+ and NYS+P+L+ groups, respectively, in comparison with the P-L- group (*p* ≤ 0.0001). The P+L+ and P+L+NYS+ groups were statistically similar to each other (*p* ≥ 0.05) (Figure 3). The other groups were statistically similar to the P-L- group (*p* ≥ 0.05).

**Figure 4:**
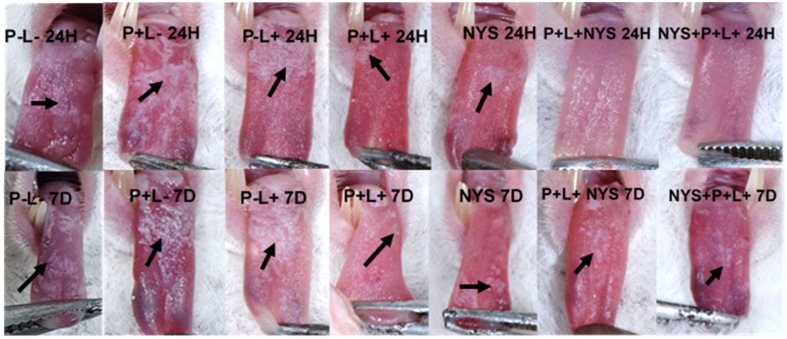
Representative images of the white or pseudomembranous patches (arrows) on the dorsum of the back of the tongue of animals from the P-L-, P+L-, P-L+, NYS and P+L+ groups after 24 h and 7 days of treatment and the absence of tongue lesions of the animals submitted to the P+L+NYS and NYS+P+L+, after 24 h of treatment.

The following images illustrate the presence of white or pseudomembranous patches on the tongue dorsum of the mice belonging to each experimental group at both of the assessed time intervals (Figure 4).

### 2.3. Histopathological Evaluation

**Figure 5:**
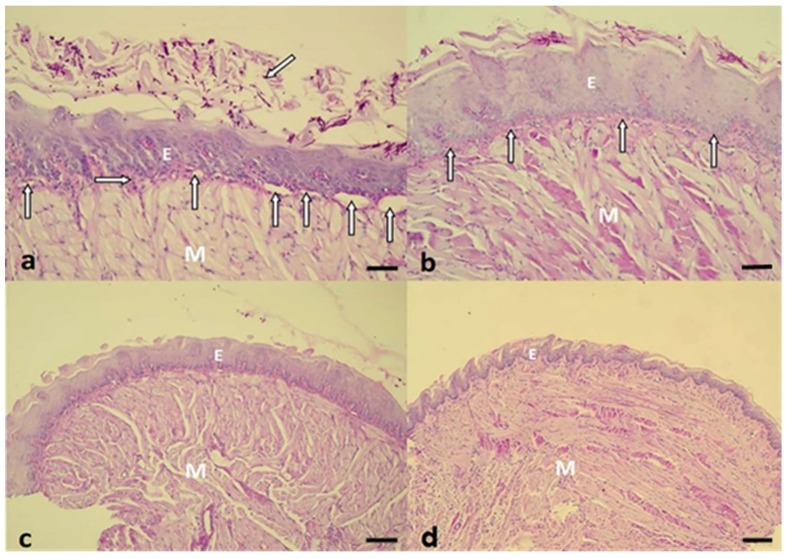
(**a**) Histological section observed in the P-L- group. There are numerous hyphaes and pseudohyphaes over the thick keratin layer (oblique arrow). Below the epithelium (E), a thin lamina propria was observed (horizontal arrow), an intense inflammatory reaction, with the presence of several dilated blood vessels (vertical arrows). The intact muscle tissue (M) is stained with Periodic Acid Schiff and Hematoxylin (PAS-H 250x). (**b**) A representative image of the NYS+P+L+ group shows the keratinized stratified acanthotic epithelium (E) covering the central portion of the tongue. The interlocking bundles of muscle tissue (M), intact epithelium, and thin layer of connective tissue (vertical arrows), exhibiting a discrete inflammatory reaction, should be noted (PAS-H 250x). (**c**) The P+L+NYS+ group presented the epithelium with lingual papillae covering the thin keratin layer. The stratified squamous epithelium demonstrated normalized histological characteristics (PAS-H 64x). (**d**) The NIC group showed a stratified squamous epithelium that masks the homogeneity of the tongue. There is a large volume of muscle tissue (M), which constitutes the major part of the tongue. All these events in the groups were observed after 24 h of treatment (PAS-H 64x). Scale bar: 20 µm

In accordance with the histopathological events, after 24 h of treatment, the histological sections of the group treated with P+L+NYS+ were similar to those observed in the healthy animals (negative infection control—NIC group), as shown by the epithelium, which presents lingual papillae covering a thin keratin layer. The stratified squamous epithelium demonstrated normal histological characteristics. The group treated with NYS+P+L+ demonstrated almost the same characteristics as those observed in the P+L+NYS group, including a greater presence of hyphae and pseudohyphae within the keratin layer. These same characteristics were also observed in the groups of animals subjected to the isolated therapies (P+L+ and NYS). However, the keratin layer was extensively contaminated with hyphae and pseudohyphaes. The animals in the P-L-, P+L- and P-L+ groups showed large amounts of hyphae and pseudohyphaes covering the epithelial tissue, which exhibited acanthosis, associated with an extensive loss of their papillae. This epithelium presents intense inflammation with the existence of mononuclear cells in the middle of dilated blood vessels. The subjacent connective tissue was formed by muscle fibers, with normal characteristics (Figure 5a–d).

**Figure 6:**
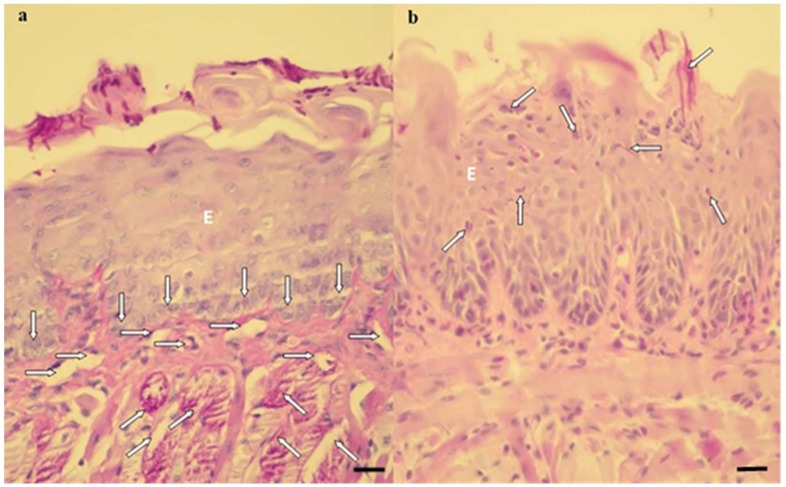
Histological sections showing an intense inflammatory reaction in the P-L-, P+L- and P-L+ groups (**a**) and a mild inflammatory reaction in the P+L+, NYS, P+L+NYS and NYS+P+L+ groups (**b**). (**a**) The cells of the basal layer of the epithelium (vertical arrows) with a thin lamina propria, present a mild inflammation. There was a predominance of mononuclear cells in the middle of a dilated blood vessel (horizontal arrows). The presence of hyphae, pseudohyphaes, and blastopores can be observed in the middle of the keratin layer, and the superficial muscular tissue was degraded (oblique arrows) (PAS-H 250x). (**b**) The keratin layer with a varying thickness and the presence of hyphae, pseudohyphaes, and high blastopore (arrows), covering the epithelial tissue (E) as well as an intact basal layer should be noted. This epithelium presents characteristics of acanthosis and does not exhibit defined lingual papillae. All these events were observed after 7 days of treatment (PAS-H 250x). Scale bar: 20 µm

Regarding the histological sections evaluated after 7 days of treatment, the observed characteristics remained the same, except in the P-L-, P+L- and P-L+ groups, which presented the muscle tissue partially degraded (Figure 6a,b).

### 2.4. Confocal Scanning Laser Microscopy (CSLM) Evaluation

**Figure 7:**
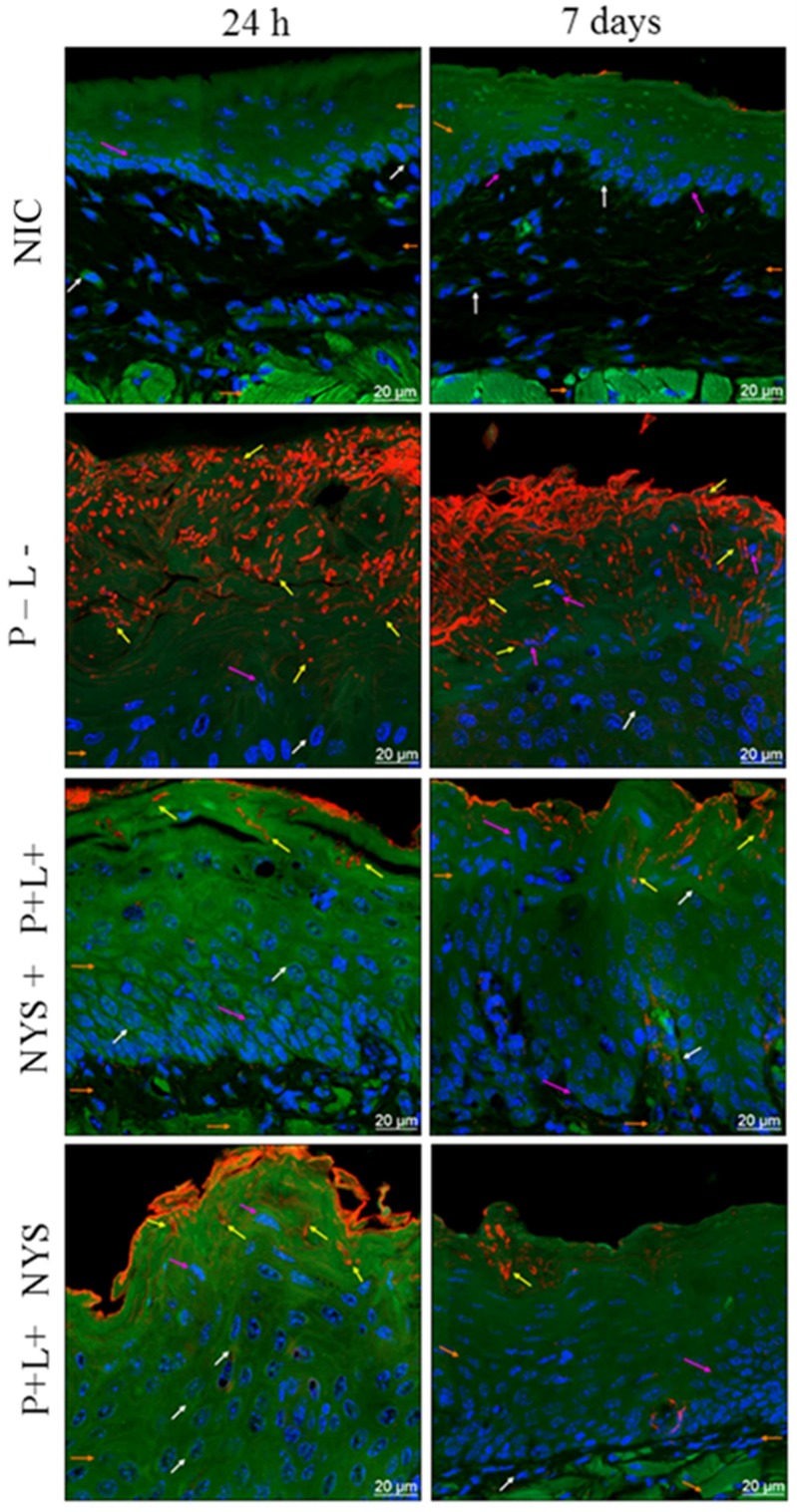
Representative images of the tongue tissues of mice with oral candidiasis by CLSM, showing nuclear morphology markings (pink arrows—nuclei labeled with Hoescht 33342); a microbial invasion (yellow arrows—*C. albicans* marked with Concanavalin 594 nm); a cytoplasmic morphology (white arrows—488 nm phalloidin-labeled cytoskeleton); an epithelial layer; connective tissue; and muscle tissue (orange arrows) for the NIC, P-L-, NYS+P+L+, and P+L+NYS+ groups, after 24 h and 7 days of treatment.

The CSLM analysis confirmed the invasion of *C. albicans* in the epithelium of the tongues of the inoculated animals (Figure 7). Twenty-four hours after the treatments, it was observed that the animals submitted to the combined therapy, P+L+NYS+, demonstrated a defined keratin layer and more organized cells in the basal layer of the epithelium, which was similar to the results for the NCI group. However, while this treatment was more effective in the inactivation of the microorganisms, it was found to have a lack of specific labelling of F-actin filaments (perinuclear region of the epithelium cells), suggesting that this alteration occurred due to the *C. albicans* infection. The group treated with NYS+P+L+ presented a larger amount of fungi in the keratin layer, thick connective tissue and a lack of specific labelling of F-actin filaments, which was similar to the results for the P-L- group. On the other hand, the images obtained from the animals treated with NYS, P-L+, P+L-, and P-L- showed microorganisms penetrating into the epithelium and a lack of specific labelling of F-actin filaments in comparison to the healthy animals (NIC group) (Figure 7).

Figure 7 also shows the images obtained after 7 days of the therapies, evidencing the presence of the microorganism in all the assessed groups. Moreover, it was possible to verify the lack of labelling of F-actin filaments, as observed after 24 h of treatment. Nevertheless, the P+L+NYS+ group still presented more organized cells in the basal layer of the epithelium in comparison to the other groups.

### 2.5. Expression of p53 and p21

**Figure 8:**
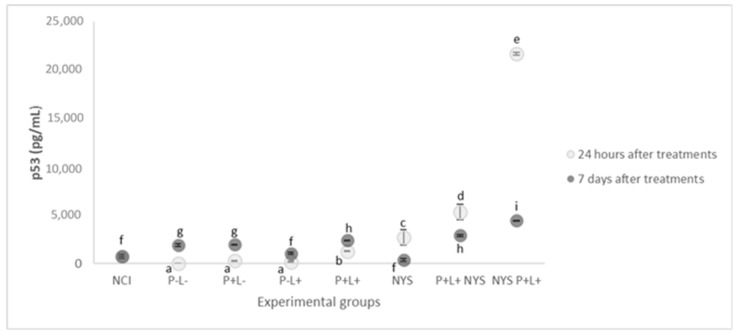
The mean values and standard deviation of the expression of p53 (pg/mL) for all of the evaluated groups. Equal lowercase letters denote a statistical similarity among the experimental groups after 24 h and 7 days of treatment (*p* ≥ 0.05).

As shown in Figure 8, the P+L+NYS+ and NYS+P+L+ groups demonstrated p53 expression levels that were statistically different from each other (*p* ≤ 0.0001) and higher than the control groups. The P+L-, P-L+ and P-L- groups showed a similar expression of p53, which was also similar to that of the NIC group (*p* = 1.000, *p* = 0.992, *p* = 0.145, and *p* = 0.629, respectively), suggesting that these treatments did not promote the production of p53 (*p* ≥ 0.05). The P-L- group did not show detectable levels of p53 expression. These results were verified after 24 h of treatment.

After 7 days of treatment, the groups that received the combined therapy (P+L+NYS+ and NYS+P+L+) exhibited reductions in p53 expression, which were statistically different from each other (*p* ≤ 0.0001) and different from the controls, P-L- and NCI (*p* ≤ 0.0001). In general, it was possible to verify a tendency in the baseline levels of p53 expression in all the experimental groups in comparison to the NIC group.

**Figure 9:**
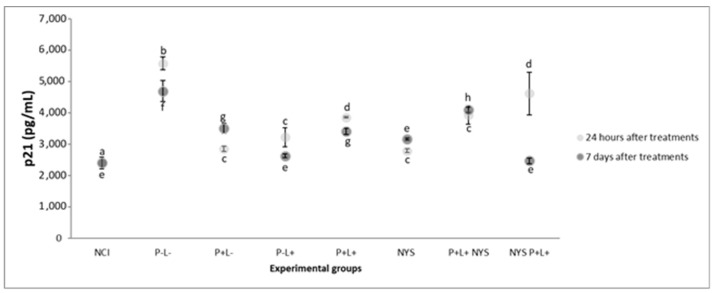
The mean values and standard deviation of the expression of p21 (pg/mL) for all the evaluated groups. The equal lowercase letters denote a statistical similarity among the experimental groups after 24 h and 7 days of treatment (*p* > 0.05).

Figure 9 shows that after 24 h of treatment, the expressions of p21 in the samples from the P+L+NYS+ and NYS+P+L+ groups were statistically similar to each other (*p* = 0.397) and different from the NIC group (*p* ≤ 0.0001). The P+L+ and NYS groups presented similar levels of p21 expression (*p* = 0.80), which were statistically different from those of the P-L- group (*p* = 0.006, and *p* ≤ 0.0001, respectively). The groups that were subjected to the isolated application of light (P-L+) and PS (P+L-) had statistically similar results (*p* = 0.890), which were different from those of the NYS+P+L+ group (*p* = 0.005, and *p* = 0.020, respectively) and the P-L- group.

After 7 days of treatment, the NYS+P+L+ and P-L+ groups presented statistically similar results in comparison with the NIC group (*p* = 0.962, and *p* = 1.00, respectively), which were different from those for the P-L- group (*p* ≤ 0.0001). The P+L+NYS+ group exhibited expression values that were similar to those observed in the P-L- group (*p* = 0.125).

## 3. Discussion

The present investigation assessed the efficacy of aPDT, applied in tandem with nystatin, in the treatment of induced OC in mice infected with *C. albicans* resistant to fluconazole. The results show that the combination of the therapies promoted a reduction of 2.6 log_10_ and 2.1 log_10_ for the P+L+NYS+ and NYS+P+L+ groups, respectively, after 24 h of treatment. The macroscopic analysis showed that the animals presented a reduction of the tongue lesions ranging from 92% to 97%. After 7 days, the observed reduction corresponded to 1.0 log_10_ and 1.1 log_10_, respectively, and the reduction of the tongue lesions was found to be 50%. According to the knowledge of the authors, the present investigation was the first to evaluate in vivo the efficacy of a combined therapy of different mechanisms of action in the treatment of induced OC in mice infected with *C. albicans* resistant to fluconazole.

Some in vitro studies have demonstrated the effectiveness of aPDT, which is associated with antifungals, in the inactivation of *C. albicans* susceptible to fluconazole [19,28]. The authors observed that miconazole increased the susceptibility of planktonic cultures of *C. albicans* to porphyrin-mediated aPDT (TMP-1363) [28]. In another investigation, it was observed that the combined application of Miconazole and aPDT, mediated by TMP-1363, was effective against biofilms of *Candida* spp., formed on acrylic resin [29]. Hsieh et al. [19] evaluated the effect of the combination of curcumin-mediated aPDT (concentrations ranging from 1 to 80 μM) with fluconazole (208 μM for 24 or 48 h) in the inactivation of planktonic cultures and biofilms of *C. albicans*. It was observed that the application of fluconazole for 48 h, followed by aPDT, using concentrations of 10 μM of curcumin, reduced the biofilm metabolism to 5%. Taken together, the data from the mentioned studies and the present study suggest that the topical application of aPDT, together with conventional antifungals, is a promising alternative for fungal inactivation, including resistant microorganisms.

In aPDT, a photosensitizer is administered topically, followed by illumination with a light source with a specific wavelength, which excites a photosensitizer and in the presence of oxygen, causes the production of cytotoxic reactive oxygen species (ROS). These ROS, produced during the reaction, may cause lethal effects on microorganisms. Oxidative stress can promote the inactivation of microorganisms by different metabolic pathways, including lipid peroxidation, the inactivation of proteins and enzymes, and the oxidation of nucleic acids [30]. In addition, it has been suggested that the produced ROS can exert oxidative stress on the polysaccharides of the extracellular matrix of biofilms, promoting its disintegration [31]. Thus, the alteration in the matrix caused by aPDT would allow for a deeper penetration of the antifungal into the layers of the biofilm, and consequently, a connection between the antifungal and the ergosterol present in the fungal membrane would be enabled [2]. This connection would facilitate the formation of channels by which the fungal cell loses the intracellular components, promoting the inactivation of the microorganism [5].

The present investigation also evaluated the effectiveness of the application of the treatments in isolation. The aPDT (P+L+) and NYS promoted reductions of 1.1 and 1.3 log_10_ in the fungal viability, respectively, after 24 h of treatment. On the other hand, Carmello et al. [17] found that five applications of PDZ-mediated aPDT were as effective as NYS in the inactivation of *C. albicans*, promoting approximately a 3-log_10_ reduction. However, the authors evaluated a strain susceptible to fluconazole. In vitro and in vivo studies have demonstrated that strains resistant to azole derivatives also showed a greater resistance to aPTD when compared to susceptible strains [16,30,32]. Dovigo et al. [32] evaluated the effectiveness of aPDT, mediated by Photogem, against planktonic cultures and biofilms of *C. albicans* and *Candida glabrata* susceptible and resistant to fluconazole and observed that the efficacy of the treatment was dependent on the species evaluated and that the strains resistant to fluconazole were less susceptible to aPDT. Similar results were observed in an in vivo investigation in which fluconazole-resistant strains (ATCC and a clinical isolate) were more resistant to PDZ-mediated aPDT (~1.96 log_10_ reduction) [16], when compared to the use of the same photosensitizer against susceptible strains (~3 log_10_ reduction) [17]. In another investigation that evaluated resistant strains, it was reported that toluidine blue-mediated aPDT was potentiated when applied with chitosan [33]. Karanja et al. [34] verified by Raman Spectroscopy an increase in the accumulation of membrane lipids in resistant strains of *C. albicans* when compared to the susceptible ones, suggesting that these lipids contributed to antifungal resistance. According to Maish et al. [35], the lipids present in the cell membrane are considered primary targets of the ROS generated in the photodynamic process. Thus, it is possible to suggest that a large quantity of ROS would be necessary to inactivate the resistant strains, and this is the one of the aspects that could justify the reduced susceptibility to aPDT of the resistant strains in relation to the susceptible ones. In the macroscopic analysis of the oral lesions, it was verified that the combined therapy (independent of the treatment sequence) promoted a reduction of ~95% in the size of the lesions present on the tongue of mice after 24 h of treatment. In these groups, the oral lesions were reduced by ~50%, after 7 days of treatment. On the other hand, the treatments, applied alone, showed similar results to those for the control groups (P-L-, P+L- and P-L+), showing white or pseudomembranous patches on the dorsum of the tongues of animals, irrespective of the assessed time interval (24 h or 7 days after the treatments). In a previous investigation, aPDT, mediated by PDZ, promoted the remission of tongue lesions after 24 h of treatment [17]. However, these authors evaluated strains susceptible to fluconazole. Thus, the divergence in the obtained results can be attributed to the type of strain evaluated and not to the protocol of the treatment.

The histological sections of the tongue tissues demonstrated normal histological characteristics similar to those of the NIC group for the animals submitted to the combined therapy (P+L+NYS+ and NYS+P+L+). The tissues presented a reduced amount of hyphae/pseudohyphae/blastopore on the keratin layer, mild inflammation in the subjacent connective tissue and intact muscle tissue. In another investigation, it was observed that the infection caused by fluconazole-resistant *C. albicans* caused an intense inflammatory response in the subjacent connective tissue [16]. These authors observed that, after aPDT (one application), the inflammatory reaction decreased from intense to mild. Regarding the control groups (P-L-, P+L- and P-L+), a large amount of hyphae/pseudohyphaes was observed to cover the epithelial tissue, which exhibited acanthosis, associated with the extensive destruction of their papillae. This epithelium presented intense inflammation, characterized by the presence of mononuclear cells in the middle of dilated blood vessels. These results were observed in the histological sections after 24 h of treatment. Seven days after the treatments, the presence of muscle fibers was verified to be partially degraded in the superficial region of the tissue. These results are in agreement with those found by Alves et al. [16], who also observed that resistant strains caused a severe inflammatory response in the subjacent connective tissue, irrespective of the type of strain evaluated (ATCC or a clinical isolate). The presence of numerous epithelial lesions, such as epithelial hyperplasia, basal layer disorganization, exocytosis, spongiosis, a loss of filiform papillae and hyperparaceratosis was verified. Okada et al. [36] reported the histological characteristics of the infection induced by clinical isolates of *C. albicans* and observed an extensive colonization, characterized by the presence of numerous hyphaes on the epithelium of the tongue, after 48 h of inoculation. In addition, the normal structure of the surrounding epithelial tongue and the papillae was damaged. Thus, our findings reinforce the idea that the inflammatory reaction found in the treated groups was associated with *C. albicans* infection and not with the protocols of treatments evaluated.

The analysis of the tongues by CLSM showed that only the animals belonging to the P+L+NYS+ group presented organized cells of the basal layer of the epithelium, which were similar to those observed in NIC group. However, the labelling of F-actin filaments was not observed, as in the epithelium of the healthy animals. Regarding the other groups, it was possible to observe the invasion of *C. albicans* in the epithelium beyond the thick connective tissue and a lack of specific labelling of F-actin filaments (in the perinuclear region of the cells) after 24 h and 7 days of treatment. It has been reported that F-actin and other cell cycle proteins were crucial for tissue restructuring [37] after injury or infectious processes [38], since the epithelial cells constitute the first line of defense against colonization by *C. albicans* [39]. Recently, Sakima et al. [40] verified that the infection caused by *C. albicans* susceptible to fluconazole, in a murine model of oral candidosis, altered the pathophysiology of the oral epithelium of animals, characterized by an increase in the production of cytokeratin (CK) 13 and 14. Twenty-four hours and 7 days after the treatments, the authors verified that the production of these CKs in the animals treated with curcumin-mediated aPDT returned to the physiological levels, similar to those observed in healthy animals. Despite the good antifungal response, the animals treated with nystatin demonstrated high levels of CK 13 and 14 production, as verified in the infected and untreated animals. Taken together, these findings and those achieved in the present study suggest that aPDT is a promising alternative for the treatment of superficial fungal infections, and irrespective of the effectiveness of antifungal therapies, the complete epithelial restructuring may also be a key factor in the prevention of fungal recolonization.

In the present investigation, the animals belonging to the combined therapy groups (P+L+NYS+ and NYS+P+L+) exhibited an increased production of p53 and p21 in the first 24 h after the treatments. Seven days after the treatments, a significant decrease in the production of p53 and p21 was identified. In accordance with the authors’ knowledge, the present study verified, for the first time, that the combined therapy stimulated the production of p53 and p21. It is important to mention that the animals with OC (P-L-) presented low levels of p53 production, indicating that, in this model of infection, there was no activation of this protein. On the other hand, the expression of p21 was observed after 24 h and 7 days of the treatments. It has been shown, in the literature, that chronic p53-independent p21 expression caused genomic instability and a deregulation of the replication licensing chemoresistance [41], and continued p21 accumulation inhibited mainly the CRL4-CDT2 ubiquitin ligase, leading to deregulated origin licensing and replication stress of DNA [41]. Therefore, it is possible to suggest that the expression of proteins p53 and p21 was regulated in a dependent manner of the combined therapy (P+L+NYS+ and NYS+P+L+). 

In summary, the P+L+NYS was more effective in the treatment of the OC of mice infected with *C. albicans* resistant to fluconazole, in comparison to the application of the same therapy in the reverse order or the application of the therapies alone. The macroscopic analysis revealed a great remission of the lesions beyond the reduction in the inflammatory response of the tongue tissues, which demonstrated normal histological characteristics. According to the American Society for Microbiology, only reductions over 3 log_10_ are considered biologically relevant [42,43,44,45]. Thus, although the results of this investigation showed significant reductions of log_10_ (CFU/mL) values, the reductions found in this investigation were lower than 3 log_10_ and are therefore not considered biologically relevant. However, it is important to emphasize that the in vivo conditions, such as the organization of microorganisms in the biofilms in the oral cavity and the immunosuppression of animals, which hampers the immune response against the infection after treatments, may affect the efficacy of aPDT. Future studies should be conducted in order to improve the efficacy of aPDT under the conditions employed in the present study.

## 4. Materials and Methods

### 4.1. Photossensitizer, LED Device

A photosensitizer (PS) substance derived from chlorin e6, Photodithazine^®^ (PDZ, VETA GRAND Co., Moscow, Russia), was used in the present investigation. This PS presents a high absorption at 660 nm. Before each experiment, PDZ (5000 mg/mL) was prepared at a concentration of 200 mg/L [46]. For this purpose, an aliquot of 20 μL of PDZ was diluted in 480 μL natrosol gel and the solution was stored at room temperature in a microtube covered with aluminum foil to avoid contact with light.

For the illumination of the tongues of animals, a handpiece containing one red LED light with an absorption peak of 660 nm (LXHL-PR09, Luxeon^®^ III Emitter, Lumileds Lighting, San Jose, CA, USA) was used. The output power of the light at the end of the apparatus was 44.6 mW/cm^2^. The samples were irradiated at 50 J/cm^2^ [46].

### 4.2. Treatments Performed

The present study was permitted by the Animal Ethics Committee of the School of Dentistry of Araraquara, UNESP (case number: 3445/2016). A total of 174 6-week-old female Swiss mice from the School of Pharmacy of Araraquara, UNESP, were employed. The animals were maintained in cages, with 5 animals per cage, under a controlled temperature (23 ± 2 °C), and they were fed with feed and water ad libitum.

A strain of *C. albicans* resistant to fluconazole (ATCC. 96901, Rockville, MD, USA) was defrosted, reactivated in an Agar Sabureaud Dextrose (SDA) medium and cultivated in RPMI 1640 at 37 °C for 16 h. Next, the cells were washed with sterile distilled water two times, and the cell suspension was standardized spectrophotometrically with an optical density (OD) of 540 nm (1.0 nm ± 0.08) (10^7^ CFU/mL) [15].

For the induction of the OC in mice, the procedure defined by Takakura et al. [47] and Carmello and co-workers [17] was performed, with some modifications. Initially, tetracycline at a concentration of 0.83 mg/mL was diluted in the animals’ water for the entire experimental period. On day 1, the animals were immunosuppressed with a subcutaneous injection of prednisolone (100 mg/kg of body weight) as follows: on days 1 and 5, the immunosuppression was performed on the animals sacrificed after 24 h of the therapies, and on days 1, 5 and 13, it was performed on animals sacrificed after 7 days of the treatments.

On day 2, *C. albicans* was orally inoculated using mini swabs, which were embedded in the standardized cell suspension and rubbed on the tongue of the animals to produce OC.

On day 7, the infection was visually identified by the presence of white or pseudomembranous patches. To perform the treatments, mice were anesthetized with ketamine (100 mg/kg of body weight) (National Pharmaceutical Chemistry Union S/A, Embu, SP, Brazil) and xylazine (10 mg/kg of body weight) (Veterinary JA Ltda., Sponsor Paulista, SP, Brazil). Next, 50 µL of the PS (200 mg/L) was applied to the dorsum of the tongue using a needleless insulin syringe and they were maintained in the dark for 20 min (pre-irradiation time). After this period, the tongues were irradiated with a dose of light, equivalent to 50 J/cm^2^ (P+L+ group). The isolated effects of the light (P-L+ group) or the PS (P+L- group) were also evaluated. The P-L- group (negative treatment control) contained animals that were inoculated with *C. albicans* but did not receive any treatment. The positive control (NYS group) animals were treated with nystatin oral suspension (100,000 I.U., Neo Química, Brazil). An aliquot of 50 µL of NYS was applied to the tongue dorsum for 39 min (time corresponding to the pre-irradiation and illumination of the P+L+ group). There were two additional experimental groups, in which the therapies were combined: The NYS+P+L+ group, containing animals that received nystatin, followed by aPDT (P+L+ group), and the P+L+NYS+ group, containing animals that received aPDT (P+L+ group) followed by NYS. These treatments were conducted in the same way as the therapies applied in isolation. After the treatments, the PS or NYS was not removed from the oral cavity of the mice. The last experimental group contained healthy animals (negative infection control—NIC group). The therapies were performed once a day for 5 consecutive days (days 7 to 11). The experimental conditions are described in Table 1.

### 4.3. Treatment Assessment

The efficacy of the therapies was evaluated by microbiological, macroscopic, histopathological and Confocal Scanning Laser Microscopy (CSLM) analyses of the lesions. In addition, the expression of p21 and p53 was evaluated.

For microbiological analysis, immediately and after 7 days of the therapies, *C. albicans* were recovered from the oral cavity of mice using sterile mini-swabs, which were soaked in tubes with 900 μL of PBS. Subsequently, serial dilutions were performed and plated on Petri dishes containing the SDA, and they were incubated at 37 °C for 48 h. The number of CFU/mL was determined.

The progression of the oral lesions was verified by taking photographs with a digital camera (Sony Cyber-Shot DSC-F717, Sony Corporation, Tokyo, Japan). Standardized photographs were taken in the beginning, after 24 h of the treatments, and after 7 days of the treatments. The extension area of the tongue lesion, presented in each photograph, was evaluated with the free edition of Image J software (free access at https://imagej.nih.gov/ij/download.html). Using this software, the percentage of the extension area of each lesion over the total area of the tongue was calculated.

All the animals were sacrificed using a lethal dose of xylazine (0.4 mL) and ketamine (0.2 mL), after 24 h and 7 days of the treatments. The tongues were removed for histopathological and CSLM analyses and to determine the expression of p21 and p53. Only a central portion of the dorsum of the tongue was used to obtain sections of 6 μm.

For the histopathological and CSLM analyses, the samples were placed in cassettes which were labeled according to the experimental group and immersed in a 10% formalin container (pH 7.4). Samples were stained with a periodic acid-Schiff reagent and hematoxylin (PAS-H) stain and observed under a microscope with 200x magnification. A histological analysis was performed by a pathologist, and the following aspects were available: the presence/absence of yeast and inflammatory infiltrate, epithelial tissue integrity and adjacent connective tissue response. The material was classified into scores: 0—absence of inflammation; 1—presence of inflammatory infiltrate; 2—moderate inflammation; 3—severe inflammation; and 4—abscess formation (ISO 7405: 1997). These scores were taken from a single examination of each experimental group and at each evaluated time interval.

For the CSLM analysis, the samples were deparaffinize and stained with Phalloidin (prepared according to the manufacturer’s instructions, Sigma Aldrich, São Paulo, Brasil), Hoechst (2 µg/mL—Thermo Fischer Scientific, Life Technologies, Rio de Janeito, Brasil) and Concanavalin A (Alexa Fluor 594) (50 µg/mL—Sigma Aldrich, São Paulo, Brasil) for the analysis of the F-actin, cell nucleus and the cell membrane of the microorganisms, respectively.

In order to evaluate the expression of p21 and p53, the samples were frozen in liquid nitrogen for 10 min and stored in a freezer at −80 °C until use. Initially, the proteins were extracted and dosed according to the Bradford methodology. Then, a Simple Step ELISA^®^ kit (Enzyme-Linked Immunosorbent Kit, Abcam, Cambridge, London, UK) was used according to the manufacturer’s recommendations. To perform the assays, the samples were positioned in the wells of the kit plate, and 50 μL of the Cocktail antibody was placed in all the wells, which were incubated at room temperature for 1 h. Next, each well was washed three times with 350 μL of a wash buffer to remove unbound material and an aliquot of 100 μL of TMB substrate was added to each well, which was incubated for 10 min. After this period, the plates were read on a 450 nm ELISA reader.

### 4.4. Statistical Analysis

Data concerning the CFU/mL of *C. albicans* were transformed into base-10 logarithms. The data were normal and heteroscedastic, so they were analyzed by a two-way ANOVA test, with the treatment performed at the two periods of time (24 h and 7 days after the treatments), followed by Games-Howell post-hoc analysis for multiple comparisons (α = 5%).

For the samples evaluated after 24 h of treatment, the percentage values related to the extension area of the tongue lesions met the assumption of normality but not the homogeneity of variance, so they were analyzed by one-way ANOVA using Games-Howell post-hoc analysis (α = 5%). Regarding the samples evaluated after 7 days of the treatments, the percentage values were normal and homoscedastic, so they were submitted to one-way ANOVA, followed by Tukey’s post-hoc analysis (α = 5%).

Regarding the histopathological evaluation, a descriptive analysis was performed for the images obtained by an optical microscope and CSLM.

The data related to the protein expressions also met the assumptions of normality and homoscedasticity. Thus, they were submitted to two-way ANOVA, followed by Tukey’s post-hoc analysis (α = 5%).

## Figures and Tables

**Table 1:** Description of the experimental groups, dosage regime and number of animals used.

Experimental Groups	Treatment Description	Number of Animals, after 24 h of the Treatments	Number of Animals, after 7 Days of the Treatments	Total Number of Animals (n)
P+L+ (aPDT)	Application of PDZ at 200 mg/L (for 20 min of pre-irradiation), followed by irradiation with LED (50 J/cm^2^) for 19 min	12	12	24
P+L-	Application of PDZ at 200 mg/L (for 20 min)	12	12	24
P-L+	Irradiation with LED, with a dose of light of 50 J/cm^2^	12	12	24
P-L-	Animals inoculated with *C. albicans*, without treatment (positive control of infection)	12	12	24
NYS	Animals treated with Nystatin oral suspension (100,000 I.U. for 39 min)	12	12	24
P+L+NYS	Application of PDZ (200 mg/L) and LED light (50 J/cm^2^) + Nystatin oral suspension	12	12	24
NYS+P+L+	Application of Nystatin oral suspension + PDZ (200 mg/L) and LED light (50 J/cm^2^)	12	12	24
NCI	Negative control of infection (healthy animals)	3	3	6
TOTAL		87	87	174

## References

[1] Martin R., Wächtler B., Schaller M., Wilson D., Hube B. (2011). Host-pathogen interactions and virulence-associated genes during *Candida albicans* oral infections. Int. J. Med. Microbiol..

[2] Gulati M., Nobile C.J. (2016). *Candida albicans* biofilms: Development, regulation, and molecular mechanisms. Microbes Infect..

[3] Brown A.J.P., Brown G.D., Netea M.G., Gow N.A.R. (2014). Metabolism impacts upon *Candida* immunogenicity and pathogenicity at multiple levels. Trends Microbiol..

[4] Sardi J.C.O., Scorzoni L., Bernardi T., Fusco-Almeida A.M., Giannini M.J.S.M. (2013). *Candida* species: Current epidemiology, pathogenicity, biofilm formation, natural antifungal products and new therapeutic options. J. Med. Microbiol..

[5] Arendrup M.C., Patterson T.F. (2017). Multidrug-resistant *Candida*: Epidemiology, molecular mechanisms, and treatment. J. Infect. Dis..

[6] Lambrechts S.G., Aalders M.C.G., van Marle J. (2005). Mechanistic study of the photodynamic inactivation of *Candida albicans* by a cationic porphyrin. Antimicrob. Agents Chemother..

[7] Donnelly R.F., McCarron P.A., Tunney M.M. (2008). Antifungal photodynamic therapy. Microbiol. Res..

[8] Bonnett R., Martínez G. (2001). Photobleaching of sensitizers used in photodynamic therapy. Tetrahedron.

[9] De Melo W.C.M.A., Avci P., de Oliveira M.N., Gupta A., Vecchio D., Sadasivam M., Chandran R., Huang Y.Y., Yin R., Perussi L.R. (2013). Photodynamic inactivation of biofilm: Taking a lightly colored approach to stubborn infection. Expert Rev. Anti Infect. Ther..

[10] Huang L., Xuan Y., Koide Y. (2012). Type I and Type II mechanisms of antimicrobial photodynamic therapy: An in vitro study on gram-negative and gram-positive bacteria. Lasers Surg. Med..

[11] Engelmann F.M., Mayer I., Gabrielli D.S., Toma H.E., Kowaltowski A.J., Araki K., Baptista M.S. (2007). Interaction of cationic meso-porphyrins with liposomes, mitochondria and erythrocytes. J. Bioenerget. Biomembr..

[12] Quishida C.C., Mima E.G., Dovigo L.N., Jorge J.H., Bagnato V.S., Pavarina A.C. (2015). Photodynamic inactivation of a multispecies biofilm using Photodithazine(^®^) and LED light after one and three successive applications. Lasers Med. Sci..

[13] Dovigo L.N., Carmello J.C., Carvalho M.T., Mima E.G., Vergani C.E., Bagnato V.S., Pavarina A.C. (2013). Photodynamic inactivation of clinical isolates of *Candida* using Photodithazine^®^. Biofouling.

[14] Carmello J.C., Alves F., Mima E.G., Jorge J.H., Bagnato V.S., Pavarina A.C. (2017). Photoinactivation of single and mixed biofilms of *Candida albicans* and *non-albicans Candida* species using Photodithazine^®^. Photodiagnosis Photodyn. Ther..

[15] Carmello J.C., Dovigo L.N., Mima E.G., Jorge J.H., Costa C.A.d., Bagnato V.S., Pavarina A.C. (2015). In vivo evaluation of photodynamic inactivation using Photodithazine^®^ against *Candida albicans*. Photochem. Photobiol. Sci..

[16] Alves F., Carmello J.C., Mima E.G.O., Costa C.A.S., Bagnato V.S., Pavarina A.C. (2018). Photodithazine-mediated antimicrobial photodynamic therapy against fluconazole-resistant *Candida albicans* in vivo. Med. Mycol..

[17] Carmello J.C., Alves F., Basso F.G., de Souza Costa C.A., Bagnato V.S., de Oliveira Mima E.G., Pavarina A.C. (2016). Treatment of oral candidiasis using Photodithazine^®^-mediated photodynamic therapy in vivo. PLoS ONE.

[18] Antoniadou A., Kontoyiannis D.P. (2003). Status of combination therapy for refractory mycoses. Curr. Opin. Infect. Dis..

[19] Hsieh Y.H., Zhang J.H., Chuang W.C., Yu K.H., Huang X.B., Lee Y.C., Lee C.I. (2018). An in Vitro Study on the effect of combined treatment with photodynamic and chemical therapies on *Candida albicans*. Int. J. Mol. Sci..

[20] Barra F., Roscetto E., Soriano A.A., Vollaro A., Postiglione I., Pierantoni M.G., Palumbo G., Catania M.R. (2015). Photodynamic and antibiotic therapy in combination to fight biofilms and resistant surface bacterial infections. Int. J. Mol. Sci..

[21] Agostinis P., Berg K., Cengel K.A., Foster T.H., Girotti A.W., Gollnick S.O., Hahn S.M., Hamblin M.R., Juzeniene A., Kessel D. (2011). Photodynamic therapy of cancer: An update. CA Cancer J. Clin..

[22] Kessel D. (2015). Autophagic death probed by photodynamic therapy. Autophagy.

[23] Martins W.K., Santos N.F., Rocha C.S., Bacellar I.O.L., Tsubone T.M., Viotto A.C., Matsukuma A.Y., Abrantes A.B.P., Siani P., Dias L.G. (2019). Parallel damage in mitochondria and lysosomes is an efficient way to photoinduce cell death. Autophagy.

[24] Abrantes A.B.P., Dias G.C., Souza-Pinto N.C., Baptista M.S. (2019). p53-Dependent and p53-Independent responses of cells challenged by photosensitization. Photochem. Photobiol..

[25] Bacellar I.O., Tsubone T.M., Pavani C., Baptista M.S. (2015). Photodynamic efficiency: From molecular photochemistry to cell death. Int. J. Mol. Sci..

[26] Castano A.P., Demidova T.N., Hamblin M.R. (2004). Mechanisms in photodynamic therapy: Part one-photosensitizers, photochemistry and cellular localization. Photodiagnosis Photodyn. Ther..

[27] Li R., Hannon G.J., Beach D., Stillman B. (1996). Subcellular distribution of p21 and PCNA in normal and repair-deficient cells following DNA damage. Curr. Biol..

[28] Snell S.B., Foster T.H., Haidaris C.G. (2012). Miconazole induces fungistasis and increases killing of *Candida albicans* subjected to photodynamic therapy. Photochem. Photobiol..

[29] Davies A., Gebremedhin S., Yee M., Padilla R.J., Duzgunes N., Konopka K., Dorocka-Bobkowska B. (2016). Cationic porphyrin-mediated photodynamic inactivation of *Candida* biofilms and the effect of miconazole. J. Physiol. Pharmacol..

[30] Kashef N., Hamblin M.R. (2017). Can microbial cells develop resistance to oxidative stress in antimicrobial photodynamic inactivation?. Drug Resist. Updat..

[31] Lopes M., Alves C.T., Raju B.R., Gonçalves M.S., Coutinho P.J., Henriques M., Belo I. (2014). Application of benzo[a]phenoxazinium chlorides in Antimicrobial Photodynamic Therapy of *Candida albicans* biofilms. J. Photochem. Photobiol. B.

[32] Dovigo L.N., Pavarina A.C., Mima E.G., Giampaolo E.T., Vergani C.E., Bagnato V.S. (2011). Fungicidal effect of photodynamic therapy against fluconazole-resistant *Candida albicans* and *Candida glabrata*. Mycoses.

[33] Huang M., Shen M., Huang Y., Lin H., Chen C. (2018). Photodynamic inactivation potentiates the susceptibility of antifungal agents against the planktonic and biofilm cells of *Candida albicans*. Int. J. Mol. Sci..

[34] Karanja C.W., Hong W., Younis W., Eldesouky H.E., Seleem M.N., Cheng J.X. (2017). Stimulated raman imaging reveals aberrant lipogenesis as a metabolic marker for azole-resistant *Candida albicans*. Anal. Chem..

[35] Maisch T., Bosl C., Szeimies R., Lehn N., Abels C. (2005). Photodynamic effects of novel XF porphyrin derivatives on prokaryotic and eukaryotic cells. Antimicrob. Agents Chemother..

[36] Okada M., Hisajima T., Ishibashi H., Miyasaka T., Abe S., Satoh T. (2013). Pathological analysis of the *Candida albicans*-infected tongue tissues of a murine oral candidiasis model in the early infection stage. Arch. Oral Biol..

[37] Xu H., Sobue T., Bertolini M., Thompson A., Dongari-Bagtzoglou A. (2016). *Streptococcus oralis* and *Candida albicans* synergistically activate μ-calpain to degrade e-cadherin from oral epithelial junctions. J. Infect. Dis..

[38] Abreu-Blanco M.T., Watts J.J., Verboon J.M., Parkhurst S.M. (2012). Cytoskeleton responses in wound repair. Cell. Mol. Life Sci..

[39] Yan L., Yang C., Tang J. (2013). Disruption of the intestinal mucosal barrier in *Candida albicans* infections. Microbiol. Res..

[40] Sakima V.T., Barbugli P.A., Cerri P.S., Chorilli M., Carmello J.C., Pavarina A.C., Mima E.G.O. (2018). Antimicrobial photodynamic therapy mediated by curcumin-loaded polymeric nanoparticles in a murine model of oral candidiasis. Molecules.

[41] Galanos P., Vougas K., Walter D., Polyzos A., Maya-Mendoza A., Haagensen E.J., Kokkalis A., Roumelioti F.M., Gagos S., Tzetis M. (2016). Chronic p53-independent p21 expression causes genomic instability by deregulating replication licensing. Nat. Cell. Biol..

[42] Nakonieczna J. (2017). Comment on Effectiveness of antimicrobial photodynamic therapy (AmPDT) on Staphylococcus aureus using phenothiazine compound with red laser. Lasers Med. Sci..

[43] Cieplik F., Tabenski L., Buchalla W., Maisch T. (2014). Antimicrobial photodynamic therapy for inactivation of biofilms formed by oral key pathogens. Front. Microbiol..

[44] Taraszkiewicz A., Grinholc M., Bielawski K.P., Kawiak A., Nakonieczna J. (2013). Imidazoacridinone derivatives as efficient sensitizers in photoantimicrobial chemotherapy. Appl. Environ. Microbiol..

[45] Kiesslich T., Gollmer A., Maisch T., Berneburg M., Plaetzer K. (2013). A comprehensive tutorial on in vitro characterization of new photosensitizers for photodynamic antitumor therapy and photodynamic inactivation of microorganisms. Biomed. Res. Int..

[46] Alves F., Alonso G.C., Carmello J.C., Mima E.G.O., Bagnato V.S., Pavarina A.C. (2018). Antimicrobial photodynamic therapy mediated by Photodithazine^®^ in the treatment of denture stomatitis: A case report. Photodiagnosis Photodyn. Ther..

[47] Takakura N., Sato Y., Ishibashi H., Oshima H., Uchida K., Yamaguchi H., Abe S. (2003). A novel murine model of oral candidiasis with local symptoms characteristic of oral thrush. Microbiol. Immunol..

